# Probing RNA structures and functions by solvent accessibility: an overview from experimental and computational perspectives

**DOI:** 10.1093/bib/bbac112

**Published:** 2022-03-25

**Authors:** Md Solayman, Thomas Litfin, Jaswinder Singh, Kuldip Paliwal, Yaoqi Zhou, Jian Zhan

**Affiliations:** 1 Institute for Glycomics, Griffith University, Parklands Dr. Southport, QLD 4222, Australia; 2 Signal Processing Laboratory, School of Engineering and Built Environment, Griffith University, Brisbane, QLD 4111, Australia; 3 Institute for Systems and Physical Biology, Shenzhen Bay Laboratory, Shenzhen 518055, China; 4 Peking University Shenzhen Graduate School, Shenzhen 518055, China

**Keywords:** solvent accessibility, SASA, RNA, probing, RNA–protein interactions

## Abstract

Characterizing RNA structures and functions have mostly been focused on 2D, secondary and 3D, tertiary structures. Recent advances in experimental and computational techniques for probing or predicting RNA solvent accessibility make this 1D representation of tertiary structures an increasingly attractive feature to explore. Here, we provide a survey of these recent developments, which indicate the emergence of solvent accessibility as a simple 1D property, adding to secondary and tertiary structures for investigating complex structure–function relations of RNAs.

## Introduction

It has been estimated that 98.8% of the human genome is not involved in the coding of proteins but instead is mostly transcribed into RNAs. That is, most RNAs are not protein-coding information carriers, but are transcribed with unknown functions. Some were found to be involved in the regulation of nearly all aspects of biological processes. Examples are protein synthesis, transcriptional regulation, RNA stability, chromosome replication and catalyzation of essential biological processes [1–3]. Moreover, novel RNAs are being constantly discovered [4] including the recent discovery of new glycoRNAs on cell surfaces [5], and novel functions for promoting spatial compartments in the nucleus [6]. RNAs, similar to proteins, execute these wide varieties of functional roles by either folding into secondary (base-pairing) or tertiary (3D) structures through base stacking interactions and hydrogen bonding across strands [2, 7]. Thus, to understand their functions at an atomic level, high-resolution structure determination and dynamic studies are the prerequisites.

Currently, RNA structure determination relies on X-ray crystallography, nuclear magnetic resonance (NMR) spectroscopy and cryogenic electron microscopy (cryo-EM). However, due to intrinsic physio-chemical properties of RNAs, it is more challenging to determine RNA tertiary structures than protein structures [8]. In fact, RNA-containing structures represent only 3% of what has been deposited in the Protein Data Bank (PDB) [9]. These RNAs with solved structures represent only a tiny fraction (<0.001%) of known non-coding RNAs (ncRNAs) [10].

The lack of tertiary structural knowledge for most RNAs has led to the development of an array of chemical techniques for probing secondary and tertiary structural properties [11]. The advancement of high-throughput sequencing (HTS) technology has made it possible to investigate RNA structures in different environments at the genome scale. These techniques employ enzymes or small molecules to react with nucleotides in a strand or base-specific manner, and reaction products can be inferred from sequencing data [11–13]. Such *in vivo,* genome-scale, structural interrogation has advanced our understanding of RNA–RNA and RNA–protein interactions as well as the overall RNA structure–function relationships [14, 15].

Meanwhile, the cost and labour-intensive nature of the above experimental techniques have inspired the development of many computational modelling approaches [16, 17]. Most of these approaches aimed to find a secondary structure with the minimum free energy (MFE), in line with the theory that an RNA molecule, like a protein, is likely to reside in a MFE state. Examples are mfold [18], the first MFE-based RNA secondary structure prediction tool, the Vienna RNA package [19], UNAfold [20] and RNAstructure [21]. Recent advances in deep learning allow end-to-end prediction of RNA base-pairing structures with improved performance not only in canonical base pairs but also in pseudoknots and non-canonical base pairs associated with tertiary interactions [22–25].

One important, simplified measure for RNA structural properties is solvent accessible surface area (SASA). Unlike RNA secondary structure, which is made of mostly local canonical base-pairing contacts, SASA is a direct tertiary-structure descriptor, indicating whether a base is exposed to or buried from the solvent. Those bases exposed to the solvent will more likely participate functional activities such as binding and catalysis. Indeed, SASA has been found useful for characterizing the conformational change due to the binding with other molecules [26], identifying hotspots at protein–RNA interfaces [27], and analyzing the structural differences among denatured, *in vitro,* and *in vivo* states [2, 28] as well as identifying disease-causing genetic variations [29]. Here, we will provide an overview of recent progresses in experimental and computational approaches to RNA SASA as it has been an overlooked area of research. Other recent reviews on studies of RNA secondary or tertiary structures can be found elsewhere [14, 22, 30, 31].

## Solvent accessible surface area: the basics

**Figure 1:**
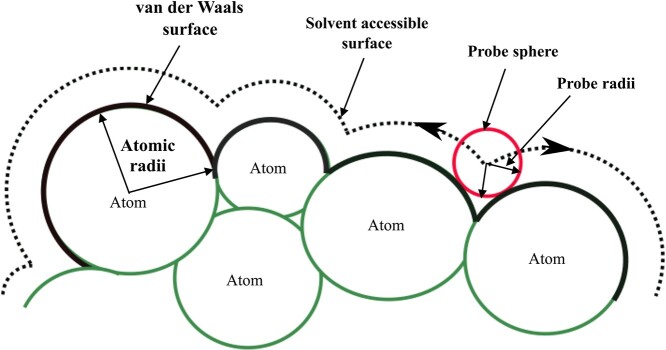
Depiction of the solvent accessible surface compared to the van der Waals surface. The rolling of a spherical probe (red) with a diameter of 1.4 Å (to approximate a water molecule) generates the solvent accessible surface.

Solvent accessibility is defined as the accessibility of residues of a macromolecule to the solvent. SASA is calculated by ‘rolling’ a probe sphere (e.g. a hypothetical spherical water) over the van der Waals surface (VdW) of a biomolecule, which is a surface created by spheres in atomic van der Waals radii around the atoms of a given molecule (Figure 1). The surface area of a residue can be visited by the ‘water’ probe on the top of the van der Waals surface is defined as the SASA. The mathematical formula for calculating SASA defined by Lee and Richards [32] is as follows:

$$ \mathrm{SASA}=\sum \left[R/\sqrt{\left({R}^2-{Z}_i^2\right)}\right]{L}_i\times D $$

$$ D=\Delta Z/2+\Delta ^{\prime }Z $$

where SASA of an atom is the area on the surface of a sphere of radius *R*, the radius *R* is given by the sum of the VdW’s radius of the atom and the selected radius of the solvent molecule, ${L}_i$ is the length of the arc drawn on a particular section $i$, ${Z}_i$ is the perpendicular distance from the centre of the sphere to the section $i$, $\Delta Z$ is the spacing between the sections, and $\Delta ^{\prime }Z$ is $\Delta Z/2$ or $R-{Z}_i$, whichever is smaller. The summation is over all arcs drawn for a specific atom. The accessible surface area is further divided by $4\pi{R}^2$ and multiplied by 100 to determine the accessibility.

$$ \mathrm{Accessibility}=100\times \mathrm{SASA}/4\pi{R}^2 $$

## Applications of RNA solvent accessibility

The adoption of particular RNA structures is required for their functional varieties. The specific structures are linked to the precise biological functions and cellular processes such as gene expression regulation [33], transcription [34], translation [35], proteins and small-molecules binding [36], RNA stability and their degradation [37]. The inherent ability of RNA to fold into complex secondary or tertiary structures creates binding pockets for metabolites (e.g. coenzymes, vitamins) and cations (e.g. Mg^2+^, K^+^), which cause the structural transition to playing different cellular roles [38, 39]. Similarly, disruption of a RNA structure, such as a mutation, can alter proper folding or RNA–protein interactions and prevent RNA metabolism, which lead to the development of different diseases [40–42]. Therefore, various computational and experimental approaches have been developed to decipher the RNA structural properties.

SASA is a 1D representation of the tertiary structure of a macromolecule such as RNA or protein. SASA values of proteins were first found to be significantly correlated with hydrophobicity, molecular weight, radius of gyration, intermolecular hydrogen bonding and transfer-free energy. For example, Chothia [43] established a linear association between SASA and hydrophobicity of non-polar side chains in amino acid residues. Upon protein folding, there was a loss of SASA as non-polar residues become packed inside the protein core, which contributes to its stability. Therefore, the average loss of SASA of an amino acid residue provides a measure of hydrophobicity of that amino acid [44]. A linear correlation was also found between the SASAs and protein molecular weights. Islam *et al.* [45] calculated the SASA values of 58 individual proteins (39 monomeric and 19 dimeric) from crystal structures and showed a direct relationship between SASA and molecular weight in both monomeric and dimeric proteins. Ooi *et al.* [46] successfully employed SASA for estimating the enthalpy and heat capacity of hydration, in addition to the free energy. Efimov *et al.* [47] showed that the solvent accessibility of donors and acceptors to water molecules determines the intramolecular hydrogen bonding in proteins: there is an inverse relationship between the number of H-bonded side chains in proteins and the SASA of their donor and acceptor groups.

In addition, SASA can be naturally applied to measure conformational transitions induced by binding interactions. Bustamante *et al*. [48] developed single and multivariate logistic regression models indicating that increased SASA of an amino acid is associated with its polymorphism regardless of its size, physicochemical properties and secondary structural element. Mukherjee *et al.* [26] compared the SASAs of 126 protein–RNA complexes between bound and unbound states. They found that both binding partners gained accessibility at the interface region upon close-to-open transitions, whilst accessibility lost in open-to-close transitions. Interestingly, binding with RNA-binding proteins (RBPs) leads to reduced accessibility at non-interface regions for the majority of RNAs but not for proteins, indicating improved structural stability of RNAs upon binding with proteins.

Unlike proteins, RNAs are only made of four nucleobases with two different sizes: purine (adenine and guanine) and pyrimidine (cytosine and uracil) bases, which have a maximum accessible surface area of 300 and 260 Å^2^, respectively [49]. Weeks and Crothers demonstrated the usefulness of probing RNA structural surfaces by performing chemical acylation experiments on exposed bases with diethyl pyrocarbonate (DEPC) [50]. They revealed the role of accessible major groove at duplex termini in molecular recognition, catalysis and structural folding. This work highlighted the importance of investigating solvent accessible surface of RNA and triggered the development of many other probes for investigating genome-scale structural characterization and their associations with RNA functions [14]. Computationally, predicted SASA values have been found positively correlated with minor allele frequencies for both coding and noncoding RNA regions [29]. That is, genetic mutations at buried RNA sites are associated with a lower minor allele frequency and are subsequently more likely to be disease causing. Furthermore, the SASA of tRNA, rRNA and mRNA revealed temperature adaptation of RNA structures in hyper-thermophilic species [51].

Thus, considering these vital relationships with structural and functional properties, SASA has been considered as a key parameter to study for macromolecules. For RNA SASA studies, both experimental and computational methods have been developed.

## RNA structure probing reagents and their readout methods

Due to the dynamic nature of RNA structures and the challenges in RNA structure determination, different enzymatic or chemical probing methods have been developed to interrogate RNA structures, some of which are at a single nucleotide resolution. They characterize RNA structures genome-wide across many species and conditions [14]. These methods are used to identify the ligand-binding sites [52], interfacial areas of RNA–RNA or RNA–protein interactions [53], canonical and non-canonical base pairs as well as RNA structural motifs including G-quadruplexes [54, 55]. In addition, these probing techniques provide the information such as solvent exposure and the tendency to be in a paired or unpaired state along the RNA chain. As enzymatic probes are sensitive to steric hindrance and restricted to *in vitro* applications, the recent methods are being developed based on either small-molecule modifications or crosslinking and proximity ligation [11, 14]. Most probes yield the information that is resulted from the combined effect of base accessibility, base-pairing and hydrogen-bonding interactions.

**Figure 2:**
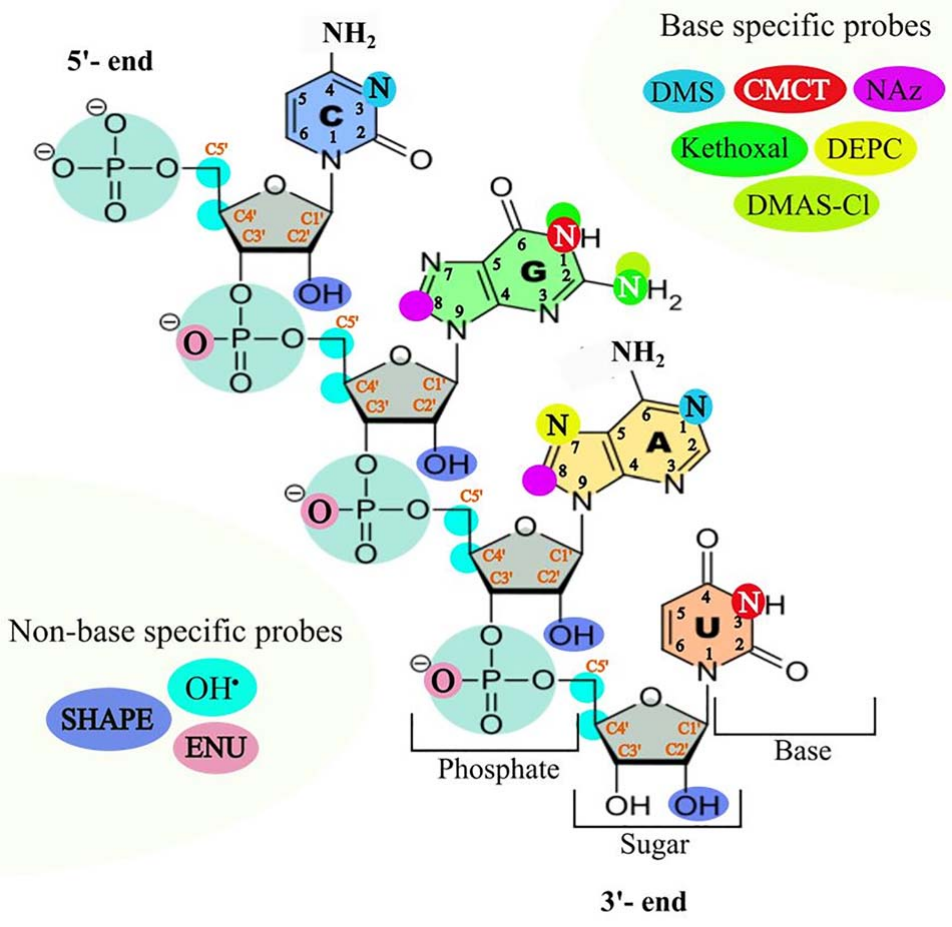
The reagents used for RNA structure probing. The probe molecules target either the sugar-phosphate backbone area or a specific base. The target sites of each probe have been shown by the respective colour. Here, SHAPE: selective 2′-hydroxyl acylation analyzed by primer extension, OH^•^: hydroxyl radical, ENU: ethylnitrosourea, DMS: dimethyl sulphate, CMCT: 1- cyclohexyl-3-(2-morpholinoethyl) carbodiimide metha-p-toluenesulfonate, NAz: nicotinoyl azide, DMAS-Cl: *N*,*N*-(dimethylamino) dimethylchlorosilane and DEPC: diethylpyrocarbonate.

There is a wide range chemical probes, which can overcome the limitations of enzymatic probing because of their small sizes. They can be either base-specific or non-base specific, which are being used to reveal the Hoogsteen and/or Watson-Crick face of the bases, as well as the sugar-phosphate backbone in RNA structures. A list of common probes employed and their target sites are illustrated in Figure 2.

One base-specific, chemical probe is dimethylsulfate (DMS) that methylates N1-A and N3-C at neutral pH. It is mainly used to recognize unpaired, accessible adenosine and cytosine nucleotides *in vitro* or *in vivo* environment. This chemical probe yields a combined effect of solvent exposure and secondary structure profile [56]. Other base specific reagents include 2- keto-3-ethoxy-butyraldehyde (kethoxal), which forms an extra ring between the primary amine positioned at C2 and N1 of the accessible unpaired guanines, 1- cyclohexyl-3-(2-morpholinoethyl) carbodiimide metha-p-toluenesulfonate (CMCT), which reacts with N3-U and N1-G of unpaired nucleotides under slightly basic conditions [57], and diethylpyrocarbonate (DEPC), which reacts with N7-A at neutral pH and after treatment with aniline. The latter allows the recognition of adenosine associated with tertiary interactions [11]. Recently, a purine nucleobase specific compound named nicotinoyl azide (NAz) has been introduced for reacting with C8 of adenine and guanine [58]. Non-base specific chemical probes interrogate the sugar-phosphate backbone. For instance, hydroxyl radicals (OH^•^) cleave the RNA backbone by reacting with the hydrogen atom at C4′ and/or C5′ ribose position(s) [59]. Ethyl-nitrosourea (ENU), an alkylating reagent, specific for the oxygen atoms of phosphate groups involved neither in tertiary interactions nor in cation coordination forms an unstable phosphate tri-ester, which causes RNA cleavage under mild alkaline treatment. It cleaves single or double stranded nucleic acids and provides the information on backbone interaction irrespective of nucleobases [60]. A number of RNA structural probes (benzoyl cyanide (BzCN), 1-methyl-7-nitroisatoic anhydride (1 M7), N-methylisatoic anhydride (NMIA), 2-methylnicotinic acid imidazolide (NAI), and 2-methyl-3-furoic acid imidazolide (FAI)) react selectively with ribose 2′-OH of flexible (usually unpaired) nucleotides. They are known as SHAPE (Selective 2′-Hydroxyl Acylation analyzed by Primer Extension) reagents and used to probe secondary structure at the profile level [61–63].

**Figure 3:**
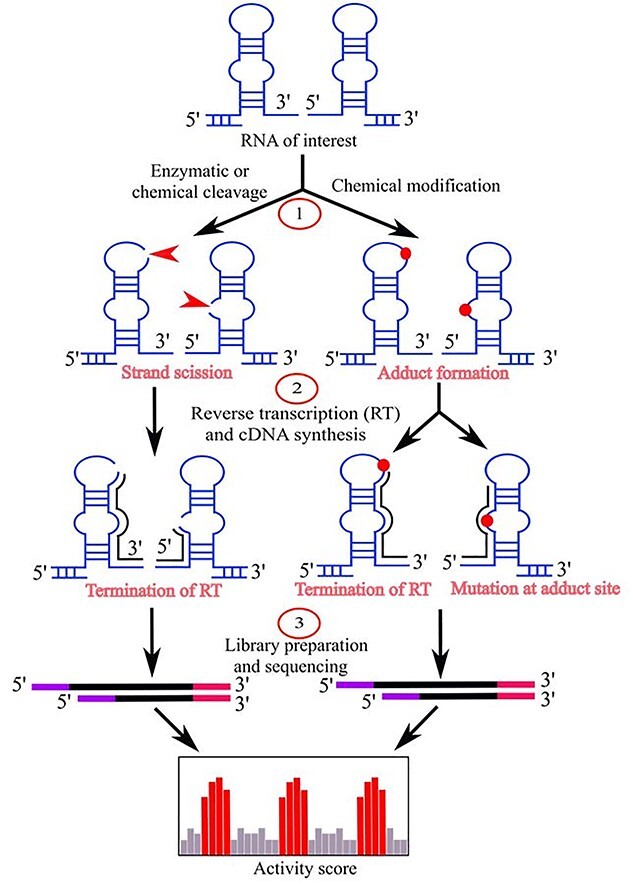
Major steps of a method with probe reactivity readout by HTS. The chemical or enzymatic probe of an RNA (*in vivo* or *in vitro*) causes strand scission (red arrow) or adduct formation (red circle) at probed sites. Then, the site information is transferred from RNA to cDNA (black line) by reverse transcription (RT) reaction either by termination of RT or mutational profiling. Finally, the HTS is performed, and the activity score is determined to map the probe reactivity along an RNA sequence.

These types of probe reagents cause the strand scission or adduct formation at a particular nucleotide that can be deciphered by proper readout methods. Recently, instead of using denaturing polyacrylamide gel or capillary electrophoresis, HTS-based readout methods are widely used (Figure 3). To address a specific biological question, suitable probes need to be chosen. Several comprehensive review articles are available for selecting the right probes [64, 65]. Here we will discuss those probes more relevant to solvent accessibility with a summary of these methods in Table 1.

**Table 1:** Experimental methods for RNA solvent accessibility probing

Methods (Year)	Probing reagents	Probing sites	Reaction conditions	Readout method	Probed RNA types	Applicability	Advantages	Disadvantages	Ref
HRF-Seq (2014)	OH^•^	C4′ and C5′ of ribose sugar	*In vitro*	HTS (RT-Stop)	RNAse P RNA, 16S rRNA	• Measuring solvent accessibility of mixture of long RNA molecules in a single tube	Includes barcoding scheme which increased the throughput of the experiment	Limited to *in vitro* study, cannot provide RNA dynamic information inside cells	[75]
						• Identifying protein ‘footprinting’ on RNA			
DMS-Seq (2014)	DMS	N1 of adenosine and N3 of cytosine	*In vitro*/*in vivo*	HTS (RT-Stop)	mRNA, rRNA	• Global monitoring of RNA structures in native conditions at single nucleotide precision	Comparative analysis of functional RNA *in vivo* and *in vitro* environments	Selectivity of DMS to solely unpaired and exposed adenosine and cytosine	[28]
						• Identification of factors affecting the structures			
LASER (2018)	NAz	C8 of purine bases	*In vitro*/*in vivo*	Denaturing gel electrophoresis	SAM-I RNA, Ligand bound SAM, rRNA, U1 snoRNA	• Monitoring solvent accessibility	Flexible method to structurally characterize RNA solvent accessibility in different environments	Conventional readout method limited the throughput of the experiment	[82]
						• Identifying rapid structural changes due to ligand binding, RNA-protein interactions and unique intracellular RNA structures			
LASER-Seq (2018)	NAz	C8 of purine bases	*In vitro*/*in vivo*	HTS (RT-Stop)	*E. coli* ribosome, K562 cell RNA, *Saccharomyces cerevisiae* ribosomes	• RNA solvent accessibility assessment	Probing of mRNA structures	Not applicable for transcriptome-wide solvent accessibility study	[58]
LASER-MaP (2018)	NAz	C8 of purine bases	*In vitro*/*in vivo*	HTS (MaP)	*E. coli* ribosome, K562 cell RNA, *S. cerevisiae* ribosomes	• Detects protein and small molecule binding sites in large RNA	Identify of binding sites and conformational changes in highly structured RNA	Not applicable for transcriptome-wide study	[58]
icLASER (2021)	NAz-N_3_	C8 of purine bases	*In vitro*/*in vivo*	HTS (RT-Stop)	SAM-I riboswitch, HeLa total RNA	• Calculates transcriptome-wide RNA solvent accessibility	Use of bi-functional probes and enrichment of probing sites	Needs extra reaction steps for enrichment process, specific to purine bases similar to other LASER methods	[84]
						• Predict protein binding sites and polyadenylation signals			

HTS: High-throughput sequencing, OH^•^: hydroxyl radical, NAz: nicotinoyl azide, NAz-N_3_: alkyl azide containing NAz.

## Experimental approaches for RNA SASA probing

### Hydroxyl radical probing

**Figure 4:**
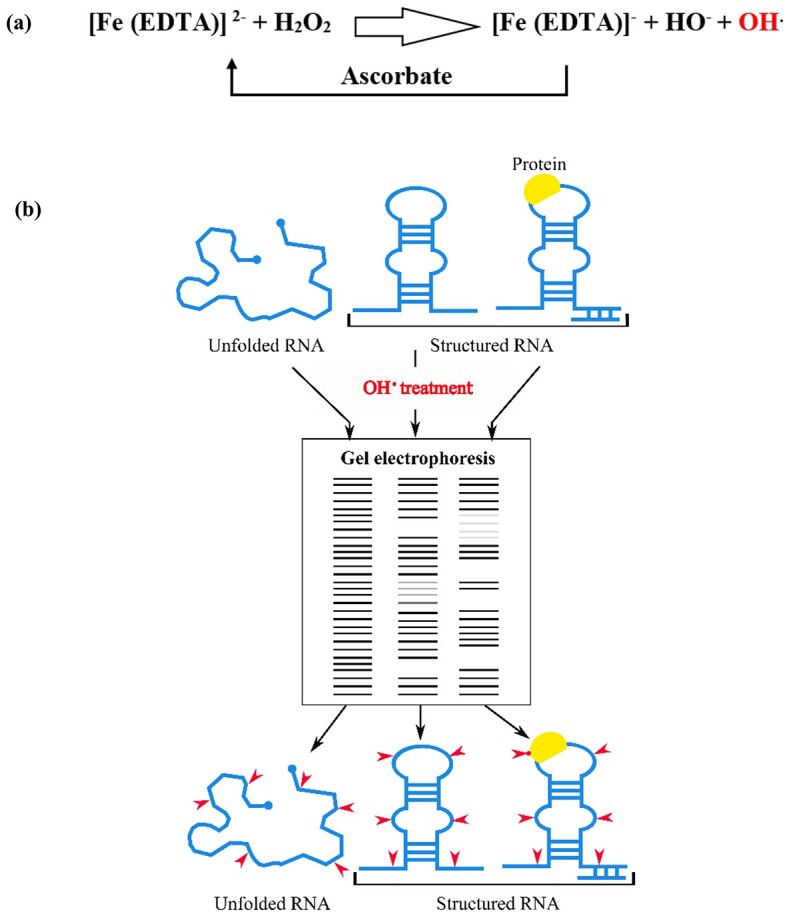
Hydroxyl radical production and probing reaction. (**A**) Fenton reaction. In this reaction, three reagents (Iron (II)—ethylenediaminetetraacetic acid, [Fe(EDTA)]_2_, H_2_O_2_ and sodium ascorbate, C_6_H_7_NaO_6_) are mixed together in the probing reaction system to create the hydroxyl radicals (OH^•^). An electron from Fe(II)-EDTA assists to reduce and cleave the O–O bond in H_2_O_2_ and generating the products: Fe(III)-EDTA, the hydroxide ion (OH^−^), and the neutral OH^•^. Sodium ascorbate reduces the Fe(III) product to Fe(II), thereby establishing a catalytic cycle and allowing low (micromolar) concentrations of Fe(II)-EDTA to be potent in cleaving RNA backbone. (**B**) Cleavage pattern is visualized by gel electrophoresis. Unfolded RNA is cleaved uniformly, whilst, only the solvent exposed area of structured RNA (including RNA–protein complex) is accessible (red arrow) to hydroxyl radicals.

Hydroxyl radical footprinting is a unique method for mapping RNA solvent accessibility directly. Hydroxyl radicals (OH^•^) are short-lived, highly reactive, oxidative species generated upon probing experiments [66]. The most widely used method of OH^•^ production is the reduction of H_2_O_2_ via the Fenton reaction [67] (Figure 4). Other techniques for generating hydroxyl radicals in nucleic-acid probing studies include reduction of solvated molecular oxygen [68], synchrotron X-ray radiolysis [69], gamma-ray radiolysis [70] or by the use of peroxonitrite [71].

There are several unique properties of hydroxyl radicals in capturing RNA solvent accessibility. Firstly, they are nucleotide and sequence independent, i.e. they induce backbone cleavage at any ribonucleotides [72] irrespective of paired or unpaired sequences with the same efficiency [68]. Secondly, they are water-like molecules with a physically defined, small radius, which reflect the degree of exposure to the solvent at the single-nucleotide resolution. This method was applied primarily to *in vitro* experiments, although it has been effectively adapted for probing assays inside the cells whereby a synchrotron X-ray beam was used to generate hydroxyl radicals [73]. Comparing the patterns of hydroxyl-radical cleavages in the presence and absence of ligands can reveal the structural details of RNA–RNA, RNA–protein or RNA–small molecules interactions (‘footprinting assays’) [59].

After hydroxyl radical probing, the cleavage sites are identified by analyzing the sample on polyacrylamide gel or capillary electrophoresis techniques (Figure 4). The cleaved products are visualized by either isotope-labelling or reverse transcribing into cDNA using end-labelled DNA primers. The achieved cleavage pattern reflects the exposure or protection area of a studied RNA molecule, which ultimately provides significant information about the interaction pattern of different units to form the final 3D structure [74]. This conventional detection process was limited to probing small RNA and one molecule at a time. Kielpinski *et al.* [75] integrated HTS technology and developed HRF-Seq for probing solvent accessibility in a high-throughput manner (Table 1). HRF-Seq had enhanced the throughput of probing readouts compared to the use of classical gel and capillary electrophoresis. They demonstrated significant correlation of ribose accessibility between the probing signals and actual accessibility calculated from the X-ray crystal structures of RNase P RNA (*r* = 0.55) and 16S *Escherichia coli* rRNA (*r* = 0.56). HRF-Seq identified protein footprints on RNA (e.g. ribosomal protein RPS21 at 723 of 16S rRNA) and used to examine the changes in RNA tertiary structures. This method made it feasible to study long and multiple RNA molecules in a single tube by using sequencing barcodes and random primers.

When it comes to determining the solvent accessibility of RNA, hydroxyl radical probing was likely to be the gold standard. It was particularly useful for measuring the accessible backbone of RNA, and commonly used to track RNA structural changes and RNA-protein interaction because of its fast rate constant. In spite of its widespread usages *in vitro*, hydroxyl radical probing in cells has been slow to take hold due to the experimental difficulties associated with employing synchrotron radiation.

### Silylation probing

Motivating from the hydroxyl radical solvent accessibility probing, Mortimer *et al.* [76] developed a novel chemical probe named *N*,*N*-(dimethylamino) dimethylchlorosilane (DMAS-Cl) that selectively reacts at the N2 position of guanosine nucleobase (Figure 2). It forms covalent adducts at the reaction sites and cleaves the RNA strand in solution. The probing sites are also readout by truncation of cDNA synthesis during reverse transcription reaction and visualized by capillary electrophoresis at single nucleotide resolution. To examine SASA, they applied this reagent on M-box RNA (*Bacillus subtilis* mgtE aptamer domain) with a known high-resolution crystal structure. They obtained a strong correlation (*r* ≥ 0.82) between the DMAS-Cl reactivity and the guanosine N2 solvent accessibility, albeit with limited, base-specific coverage of a short RNA transcript. They also observed that DMAS-Cl was reactive to N2 guanosine in a structure-selective manner in solution, more reactive when RNA lacked tertiary structure (in the absence of Mg^2+^) than the extensive higher order tertiary conformation (in the presence of Mg^2+^). Kethoxal, a carbonyl electrophile, also attacks N2 of guanosine and forms cyclic adduct with N1 position of the same base [77]. However, they found a very poor correlation (*r* ≤ 0.35) when kethoxal was employed, indicating that different chemicals may have different effects.

### DMS probing

In addition to probing secondary structural properties, DMS is also used for RNA solvent accessibility study [28]. It is highly reactive on unpaired solvent accessible N1 of adenosine and N3 of cytosine (Figure 2). DMS is permeable to cells thus allows *in vivo* monitoring of RNA structural properties and the influence of proteins on them. By integrating HTS, the first *in vivo,* genome-wide RNA structure probing method, Structure-seq, was developed based on DMS [78, 79]. Later this method was improved to ‘Structure-seq2’ by introducing enrichment of DMS probed sites by biotin–streptavidin pull down instead of polyacrylamide gel electrophoresis (PAGE) extraction [80]. Rouskin *et al.* [28] first described the use of DMS for genome-wide, RNA solvent accessibility study in their DMS-seq method. They evaluated the DMS-seq signal with the high-resolution crystal structure of yeast ribosome (18S and 25S) and observed fewer stable structures *in vivo* compared to *in vitro.* The receiver operating characteristic (ROC) curve revealed a strong relationship between *in vivo* probing signals and crystal structure models with a 90% true positive rate (solvent accessible and unpaired bases). The application of DMS-seq on mammalian cells (human foreskin fibroblasts and K562 cells) also exhibited a similar result to yeast ribosomal RNA and found that mRNAs *in vivo*, in rapidly dividing cells, are less structured than *in vitro*. They also performed genome-wide structure probing at different temperatures (30, 45, 60, 75 and 95°C) to evaluate thermal stability of mRNA. The results showed that most structures became unfolded at high temperature and RNAs with structures *in vivo* had high thermostability. At low Mg^2+^ concentration (1 mM), most RNA structures were also found unfolded *in vitro*, whilst at 2–6 mM they were similarly structured. The depletion of ATP on yeast revealed a significant increase in mRNA structures *in vivo*. In addition, the structural transition was substantially correlated (*r* = 0.54) to the interchanges between *in vivo* and *in vitro* samples upon ATP depletion. The reactivity readout of these techniques was based on RT-stop, whilst Zubradt *et al.* [81] presented DMS-MaPseq (DMS-mutational profiling) and made the method more robust and simple (Figure 3).

### Light activated probing by nicotinoyl azide

**Figure 5:**
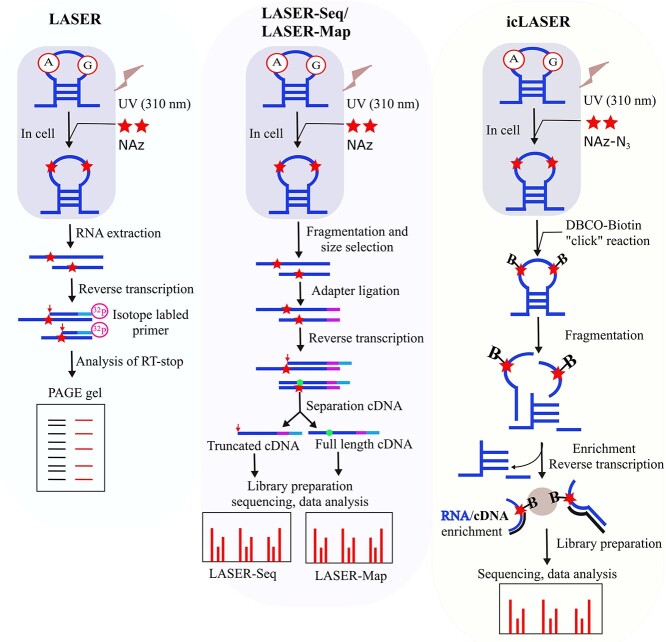
An overview of different light-activated probing methods. (**A**) LASER (light activated structural examination of RNA) employs nicotinoyl azide (NAz) as a probing reagent, which yields an adduct (red star) at C8 of purine residues (A and G). After RNA extraction, reverse transcription (RT) is performed using ^32^P isotope labelled primers, and the RT-stop (red arrow) is analyzed by denaturing PAGE. (**B**) LASER-Seq or LASER-Map also employs NAz as a probing reagent. The adapter is ligated followed by RNA extraction, fragmentation, and size selection. The RT is performed by adapter specific primers, and the cDNA synthesis either stops (red arrow) or creates a mutation (green circle) at the adduct site. Then the cDNA is fractionated, and the sequencing library is prepared. From the sequencing data analysis, RT-stop (LASER-seq) and mutational profiles (LASER-Map) are estimated to measure the probe reactivity. (**C**) icLASER uses an alkyl azide containing NAz (NAz-N_3_) instead of sole NAz as a probing agent. The dibenzylcyclooctyne biotin (DBCO-biotin) is linked at the probe site by copper-free ‘click’ reaction. After fragmentation, the probed sites are enriched by streptavidin-biotin affinity purification and RT is performed. Finally, sequencing library is prepared from the truncated cDNA and the reactive sites are obtained from sequencing data analysis.

Light activated structural examination of RNA (LASER) is the most recent and advanced technique for RNA solvent accessibility study. It was firstly developed by Feng *et al.* [82] and later, was modified and improved broadly in subsequent years (Table 1). LASER uses nicotinoyl azide (NAz) as a probing reagent, which reacts with solvent-exposed guanosine and adenosine at C-8 position of ss or dsRNA. UV light (310 nm) activates NAz molecules to become highly reactive nitrenium cations in solution and produces adducts to the solvent-exposed area of purine residues (Figure 5). Denaturing gel electrophoresis was used to readout the adduct formation and NAz reactivity. The LASER technique is sensitive to variations in solvent accessibility and informs structural transition information similar to hydroxyl radical probing. The relationship between the LASER signals and the calculated solvent accessibility of studied RNA crystal structures (SAM bound SAM-I riboswitch) was linearly correlated (*r* = 0.82). It worked with delicate sensitivity and distinguished compact structural changes in SAM-I RNA due to ligand (SAM) binding. In addition, LASER identified the key residues (A46 and A45), which are in direct contact with the ligand based on their solvent accessibility information. Probing *in vivo* 18S rRNA and U1 snRNP (complexed with proteins) revealed the ability of this method to reading RNA structures inside cells and detecting RNA-protein interactions in native environments. The use of NAz made the LASER method unique as other classical structure probing reagents (such as DMS) work by identifying single stranded regions.

Zinshteyn *et al.* [58] expanded the LASER probing method and developed LASER-Seq and LASER-MaP. These improved techniques employed the HTS for NAz reactivity readout based on RT-stop and mutational profiling, respectively (Figure 5). Both techniques were applied to ribosomal RNAs extracted from three spectrum of species (bacterial, yeast and mammalian cell) *in vitro* and *in vivo* to compare against solvent accessibility calculated by using high-resolution crystal or cryo-EM structures. The results showed that LASER-Seq and LASER-MaP were able to detect solvent accessible nucleotides in both (*in vitro* and *in vivo*) environments. However, the authors preferred LASER-Map to LASER-Seq for further experiments. They compared NAz with 1 M7, BzCN used in SHAPE-MaP [83] and DMS used in DMS-MaP [81] and found NAz produced the most mutations at G positions in all probes, and more mutations than SHAPE but less than DMS at A positions. In addition, MaP carries more information than RT-stop as it can identify multiple mutations in a single sequencing read. These benefits facilitated LASER-MaP as a potentially suitable method for structure probing with low non-specific background signals. The MaP profiling information was compared with the computed SASA at C-8 of A and G from X-ray crystal structures to produce the ROC curve. The area under the ROC curve (AUC) measured the discriminatory power of the MaP value, which were 0.75 and 0.82 for *E. coli* and *Saccharomyces cerevisiae* ribosomal RNA, respectively, and demonstrated LASER-Map as a reasonable method for quantifying the solvent accessibility. In addition, this technique was used in detecting binding sites of small molecules or ligands on RNAs and conformational changes in complex RNA in an unbiased fashion. Upon ligand binding, the nucleotides at/close to the binding sites become less accessible or protected from the probing reagents. The secondary protection of nucleotides, far away from the ligand-binding sites, may indicate the massive conformational changes because of ligand binding. The application of LASER-MaP on intact *E. coli* ribosomes incubated with EF-G (elongation factor G), in the presence or absence of non-hydrolyzable GTP analogue (GDPNP), and proline-rich antimicrobial peptide onc112 detected the conformational changes and dynamics in both paired and unpaired regions.

More recently, a bi-functional LASER probe has been developed from the same research group to measure transcriptome-wide purine C-8 solvent accessibility [84]. An alkyl azide functional group was placed on NAz as an enrichment moiety due to its light sensitivity and probing efficiency. A biotin is ligated to alkyl azide through copper-free ‘click’ reactions after the probing reaction and subjected to streptavidin coated magnetic bead enrichment (Figure 5). The improved method named as *in vivo* click LASER (icLASER), and it significantly enriched RT-stopped cDNAs. The mapping analysis revealed the enrichment had happened at A and G nucleotides as expected. This new probing technique allowed transcriptome-wide RNA solvent accessibility measurement and environment specific (in and outside of cells) RNA structures interrogation. Moreover, integrating icLASER (RNA solvent accessibility) with other reactivity-based measurements such as icSHAPE (RNA flexibility) has broadened its application to transcriptome-wide identification of RNA functional elements. Studying the IRE (iron response element) in UTR of mRNA, these methods determined the pattern of protein binding, flexibility and solvent accessibility of nucleotides based on the different chemical reactivities. The transcriptome-wide mapping of mRNA functional elements revealed the start codons are highly open and solvent accessible at the A and G of AUG, while the last two positions of stop codons were largely accessible. In addition, the solvent accessibility differences based on *in vivo* and *in vitro* accessibility profiles suggested that icLASER could be used for mapping protein-RNA interactions. The authors implemented Support Vector Machines (SVM) to generalize the icLASER and icSHAPE signals (*in vivo*) and compared with enhanced crosslinking and immunopurification (eCLIP) [85] datasets from K562 cells. The AUC analysis revealed that icLASER and icSHAPE could predict, independently, 50–70% of binding sites of 75 studied RBPs and the accuracy of icLASER prediction was higher than that of icSHAPE. The reactivity signals combined from both methods led to >90% accuracy for predicting protein occupancy on RNAs. In addition, they predicted polyadenylation signals with 87% accuracy by combining SVM with icLASER and icSHAPE reactivity profiles [86].

## RNA solvent accessibility prediction

While predicting protein solvent accessibility has been around for three decades [87, 88], the history of predicting RNA solvent accessibility is relatively short. To our knowledge, there are only three machine-learning-based methods (RNAsnap, RNAsol, and RNAsnap2) developed so far (Table 2).

**Table 2:** Computational predictors of RNA solvent accessibility

Tools (Year)	Used algorithm	Solvent accessibility prediction	Advantages	Disadvantages	Webserver link	Ref.
RNAsnap (2017)	Support-Vector Machines	Protein-bound RNA	Shows positive correlation of mRNA solvent accessibility with experimentally calculated data Predict SASA of the disease-causing nucleotides at single nucleotide variant Differentiate SASA of coding and non-coding region of RNAs and define their functional roles	Has significantly low performance of SASA prediction for protein-free RNAs	Discontinued with RNAsnap2	[29]
RNAsol (2019)	LSTM neural networks	Protein-bound RNA and protein free RNA	Perform better than RNAsnap for predicting SASA of protein-bound and protein-free RNAs	Cannot predict accurate RSA of nucleotides involved in secondary and tertiary structural motifs	https://yanglab.nankai.edu.cn/RNAsol/	[89]
RNAsnap2 (2020)	Dilated convolutional neural network	Protein-bound RNA and protein free RNA	Can predict SASA with or without sequence profiles Able to calculate RSA of nucleotides involved in secondary-structural-motifs	Performance decrease with the increase of RNA sequence length Low performance in RSA prediction of nucleotides involved in tertiary structural interactions	https://sparks-lab.org/server/rnasnap2/	[90]

mRNA, messenger RNA; SASA, accessible surface area; RSA, relative solvent accessibility.

**Figure 6:**
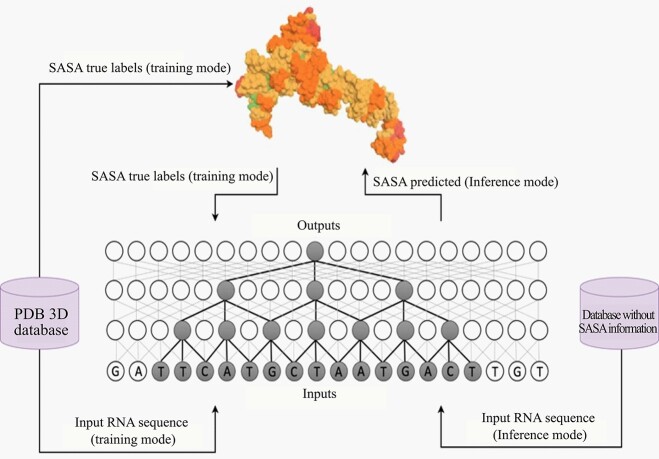
Generalized model architecture of machine learning-based RNA solvent accessibility prediction. Machine learning models are first trained with sequence-derived information as input and true SASA labels from a training set for training model parameters. The model trained then can be used for inference (prediction).

Figure 6 shows the general workflow of these computational machine-learning-based SASA predictors, which operate in two modes: training and inference. During the training mode, they employ a database of known SASA information (usually from the protein data bank, PDB) to learn machine learning model parameters through an iterative process. RNAsnap, RNAsol and RNAsnap2 methods differs from each other in terms of the machine-learning model used for prediction. RNAsnap, RNAsol and RNAsnap2 deployed support-vector-machine (SVM), unidirectional long-short-term-memory (LSTM) and dilated convolutional neural network, respectively.

During the inference mode, these models take sequence-derived information to predict RNA solvent accessibility. Inference mode is computationally less expensive as compared to the training mode. Next paragraphs discuss individual methods in more details in terms of the datasets used and their performance on the test sets.

In 2017, Yang *et al.* [29] developed RNAsnap that was dedicated to prediction of RNA solvent accessibility. It was a machine-learning based method trained on protein-bound RNA structures. This method utilized nonredundant, high-resolution (<3.0 Å) 89 RNA structures complexed with proteins as a training dataset (TR89), and evaluated on 48 protein-free RNAs (CN48) and 44 RNA complexed with proteins as a test set (TS44). The authors developed two separate SVM models named RNAsnap-seq and RNAsnap-prof by inputting either the query RNA sequence only or the sequence profile from multiple sequence alignment against the query sequence, respectively. The Pearson’s correlation coefficient (PCC) between actual and predicted solvent accessibilities calculated from RNA structures was used for measuring the models’ accuracy. RNAsnap-seq achieved PCC of 0.595 for the training dataset (TR89) and 0.538 for the independent test set (TS44), but only 0.225 for 48 protein-free RNA structures (CN48). The corresponding PCC values for RNAsnap-prof were 0.655, 0.630 and 0.228, respectively. Thus, it only succeeded to gain a decent performance for protein-bound RNAs but not for protein-free RNAs. The application of this method to 6178 mRNAs showed its positive correlation to *in vivo* mRNA accessibility determined by the DMS probing technique, not to *in vitro*. It also showed a positive correlation (PCC > 0.8) between the projected SASA of the mutation site of single nucleotide variant (SNV) with the minor allele frequency in the 1000 Genomes Project, which indicated damaging effects of inaccessible RNA core structures. A study of 15 642 gene transcripts with RNAsnap revealed introns are more solvent-exposed than exons. The application of this tool to monitor temperature adaptation of rRNA, tRNA, and mRNA from 200 bacterial species showed that rRNA and tRNA were more exposed to solvent but remained structured in hyper-thermophilic bacteria [51].

RNAsol was developed to improve over RNAsnap by using a relatively larger training set and a deep learning technique [89]. This method was built on improved sequence profiles from covariance models and trained with the long short-term memory (LSTM) neural networks. RNAsol was compared to RNAsnap by re-building the prediction model with the same training dataset (TR89, protein-bound RNA) with the optimized parameters of RNAsol. The comparison on the independent test sets (TS44, protein-bound; CN48, protein-free RNA) showed that RNAsol achieved the PCC of 0.43 and 0.26, respectively, an improvement over the RNAsnap tool (PCC of 0.34 and 0.11, respectively). The accuracy of the protein-free CN48 set was lower than the protein-bound TS44 dataset in both tools as the train set (TR89) was only protein-bound. However, when the training set included a mix of protein-bound and protein-free RNAs (TR120), the PCC of CN48 increased to 0.46 from 0.26. The authors claimed the use of Infernal-based profiles and predicted single sequence (SS)-based secondary structure information contributed for the better performance of RNAsol instead of using BLASTN-based profiles in RNAsnap. The application of RNAsol on ‘Bacterial Ribonuclease P Holoenzyme Complex’ (protein-bound) and ‘Specificity domain of Ribonuclease P of the A-type’ (protein-free) showed a reasonable correlation between the predicted and real SASA values (PCC of 0.47 and 0.46, respectively).

RNAsnap2 was developed in 2020 [90]. The accuracy and precision of solvent accessibility prediction has significantly improved over the above-mentioned two methods. RNAsnap2 showed 11% improvement in median PCC (0.539) and 9% in mean absolute errors (MAE) (32.80) for the identical test dataset used in RNAsol (TS45). Moreover, there was a greater improvement (22% median PCC, 0.509) in case of 31 non-redundant newly deposited protein-free RNA chains (TS31), which was independent from the training and the test datasets. A single-sequence version of RNAsnap2 (SingleSeq) achieved a comparable performance to RNAsol for TS45 (PCC = 0.500) and a better performance for TS31 (PCC = 0.483), despite that it did not use evolutionary information. RNAsnap2 differs from RNAsol by using predicted base-pair probabilities from LinearPartition and a dilated convolutional neural network architecture, instead of using predicted secondary structure from RNAfold (MFE) and unidirectional LSTM neural networks. Unlike RNAsol, RNAsnap2 offers two versions (with or without evolution sequence profiles). However, both tools are still facing challenges for predicting SASA for long RNAs (>300 nt) because of the lack of training data for long sequences. While RNAsnap2 can performed reasonably well for identifying relative SASA (RSA) of bulge, stem, hairpin, internal, multi, and exterior loop nucleotides (PCC = 0.409–0.598), a poor performance was observed for predicting RSA of nucleotides involved in tertiary interactions such as pseudoknot base pairs and base multiplets. The direct comparison of RSA with high-resolution crystal structures of non-coding Y RNA, adenovirus virus-associated RNA, and Glutamine II riboswitch also indicated better performance (PCC of 0.83, 0.55 and 0.44, respectively) of RNAsnap2 than the other two tools. A detailed comparison of the three tools is shown in Table 2.

**Figure 7:**
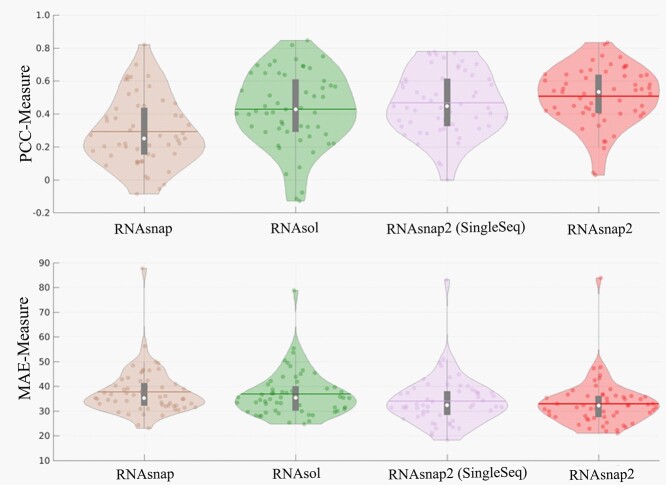
Comparative performance of the SASA predictors with the same data set. Violin plot of PCC and MAE of SASA predicted by RNAsnap, RNAsol, RNAsnap2 (SingleSeq) and RNAsnap2 on benchmarking test of 57 PDB RNAs non-redundant from all predictors training data according to CD-HIT-EST at lowest allowed sequence identity cut-off of 0.8 followed by BLASTN with *e*-value of 10 against their training data. In the Violin plot: white dot represents the median; the thin horizontal red line represents mean; the thick and thin grey bar in the centre represents the interquartile range and 1.5× interquartile range; the curve on either side of grey line shows the distribution of data using kernel density estimation; wider the curves around grey lines, higher the probability of data points lies in that region and *vice versa*.

To further evaluate these models, we prepared a benchmarking test set of 57 high-resolution (<3.5 Å) X-ray structures from PDB. This test set is non-redundant from training data of all three predictors according to CD-HIT-EST at lowest allowed sequence identity cut-off of 0.8 followed by BLASTN with *e*-value of 10. Figure 7 shows distribution of PCC and MAE of predicted SASA for 4 methods including RNAsnap2 (SingleSeq). On this test set, RNAsnap2, RNAsnap2 (SingleSeq), RNAsol and RNAsnap achieve median PCC of 0.53, 0.44, 0.42 and 0.25, respectively. Similar trends were also observed in terms of MAE with RNAsnap2, RNAsnap2 (SingleSeq), RNAsol and RNAsnap, which were 32.23, 32.28, 35.31 and 35.22, respectively (Figure 7).

## Future perspective and conclusions

The main experimental approaches for RNA solvent accessibility study include hydroxyl radical probing, LASER, and its improved versions (LASER-Seq, LASER-MaP and icLASER). The integration of HTS technology, similar to other advanced RNA structure probing methods, has allowed transcriptome-wide solvent accessibility studies. However, the reactivity readout procedures are based on either the RT-stop at cleavage points or mutational profiling at the adduct formation sites (Figures 3 and 5). The RT reaction, which utilizes the random primers and the reverse transcriptase enzyme, has several drawbacks. For example, random priming results in short and truncated sequences because of internal priming, which causes overrepresentation of copy numbers in subsequent round of amplification. In addition, reverse transcriptase suffers from non-specific drop-off without completing the reaction [91]. These pitfalls lead to spurious truncated sequences and subsequently cause false positives for probe-reactive sites. As a result, current methods often require a high-sequencing depth to improve the accuracy of transcriptome-wide probing profiles. A bi-functional probing reagent used in icLASER has been developed to alleviate these issues. One potential solution is to directly block (or link) the probed ends with a known linker sequence prior to the RT reaction with specific primers and counting the first nucleotide immediately after the linker as the probed sites. One advantage of LASER methods is their ability to probe in living cells. However, the current probe reagents (NAz, NAz-N_3_) are only purine base specific. Thus, the development of a new method that can cover all nucleobases or integration with other probing techniques may be needed to overcome this limitation.

Computationally, only three methods were developed for RNA solvent accessibility prediction. These methods obviously can be improved further given constantly improving deep learning techniques and increasing availability of training data. However, the major challenge for RNA solvent accessibility prediction remains the relatively small number of RNA structures that are available in the protein databank. There is a risk of over-training if not trained appropriately. Thus, transcriptome-wide experimental data may be used for initial training, following by transfer learning with SASA from known structures. Moreover, a newly developed automated tool for RNA sequence profile and correlated mutation analysis [92] can be employed for new features to better capture evolution information.

Currently, applications of predicted or experimentally estimated RNA SASA values remain limited. The analysis of RNA SASA provides information on possible solvent-exposed interaction sites, disease-causing mutations, structured and unstructured areas based on the pattern of solvent accessibility. For example, RNAsnap [29] detected a positive link between the minor allele frequency of a SNV and the predicted SASA value of the mutation site of the SNV. RNAsnap also shows adaptation of rRNA, tRNA and mRNA structures for bacterial species living in different temperature environments [51]. Experimentally, integrating HTS technology, HRF-Seq improved the throughput of probing study and identified footprinting of ribosomal proteins on 16S rRNA based on solvent accessibility [75]. DMS-Seq revealed that RNAs extracted from various organisms (e.g. yeast, bacteria and human cells) are structurally different between *in vivo* and *in vitro.* This method also investigated the RNA structural stabilities at different temperatures, Mg^2+^ concentrations, and in the presence or absence of ATP [28]. Using solvent accessibility, LASER methods (LASER-Seq/LASER-Map) located ligand-binding sites and distinguished the ribonucleotides involved in the RNA–ligand interactions [58, 82]. The most recently developed bi-functional probing reagent-based method, icLASER, permitted transcriptome-wide RNA solvent accessibility study. Linking with other probing methods such as icSHAPE, this technique has expanded the applications to identify genome-wide RNA functional elements and determine protein binding, flexibility and polyadenylation signals [84].

In future, we expect that high-throughput experimental SASA probing data will be employed as a part of training data for initial learning to supplement the scarcity of RNA structural data for secondary and tertiary structure prediction, as exemplified by the use of approximate secondary structure database for transfer learning in SPOT-RNA [23]. The profiles of unpaired/paired bases generated by SHAPE and DMS experiments have already been used for improving secondary structure prediction [93] and tertiary structure prediction as a part of integrative modelling [94]. Thus, the results of SASA from LASER-Seq or icLASER experiments as well as from computational prediction will be likely integrated in 3D structure modelling in near future, similar to the use of SASA in *ab initio* protein structure prediction [95], template-based structure modelling [96] and native structure discrimination [97].

**Key Points**

- This work surveys current experimental techniques and computational methods for characterizing RNA solvent accessibility, an underexplored area of research.
- Solvent accessibility is a simple 1D measure for characterizing RNA 3D structure, complementary to 2d RNA secondary structure.
- The experimental techniques include hydroxyl radical, DMS and LASER probing and computational predictors are machine-learning-based RNAsnap, RNAsol and RNAsnap2.
- RNA solvent accessibility will be useful for improving structure prediction and discovering potential functional sites.

## Data Availability

The data underlying this article will be shared on reasonable request to the corresponding author.

## Author Biographies

**Md Solayman** is a PhD student at Institute for Glycomics, Griffith University. His research interests focus on RNA structure probing, protein biochemistry, and bioinformatics.

**Thomas Litfin** received his Ph.D. under Prof. Yaoqi Zhou’s supervision from Griffith University and joined in Institute for Glycomics as a Research fellow. His research interests include deep learning and structural bioinformatics.

**Jaswinder Singh** is a Ph.D. student in Signal Processing Lab, Griffith University. His research interests focus on artificial intelligence, machine learning and bioinformatics.

**Kuldip Paliwal** joined Griffith University in 1993 as Professor (Chair, Communication/Information Engineering) in the School of Microelectronic Engineering and established the Signal Processing Laboratory. His research area includes signal processing, speech processing, machine learning and bioinformatics.

**Yaoqi Zhou** is a senior principal investigator of the Institute for Systems & Physical Biology, Shenzhen Bay Laboratory, China. Previously, he worked at State University of New York at Buffalo, Indiana University at Indianapolis, and Griffith University. His research interests focus on computational biology, bioinformatics, biophysics and molecular biology.

**Jian Zhan** is an investigator at Institute for Systems & Physical Biology, Shenzhen Bay Laboratory, China. His research interest includes RNA structure biology, protein engineering and biophysics.
